# Mucoepidermoid carcinoma of unknown primary in the head and neck: a case report and review of the literature

**DOI:** 10.1017/S0022215124002147

**Published:** 2025-06

**Authors:** Kieran Chalmers, Phillip Staibano, Michael K. Gupta, Michael Au

**Affiliations:** 1 Division of Otolaryngology–Head and Neck Surgery, Department of Surgery, McMaster University, Hamilton, ON, Canada; 2 Michael G. DeGroote School of Medicine, McMaster University, Hamilton, ON, Canada; 3 Department of Health Research Methods, Evidence, and Impact, McMaster University, Hamilton, ON, Canada

**Keywords:** clinical management, mucoepidermoid carcinoma, head and neck cancer, unknown primary neoplasm

## Abstract

**Objective:**

Mucoepidermoid carcinoma of unknown primary (MEC-UP) in the head and neck is a rare presentation of the most common salivary gland cancer. Cancers of unknown primary sites often have poorer prognoses than similar cancers with known primary. Few cases of MEC-UP have been reported; therefore, the objective of this report is an overview of the diagnosis and management of MEC-UP.

**Methods:**

We present two patients with low-grade MEC-UP at a high-volume tertiary care institution in Ontario, and a database search returning 1560 citations of which five studies with seven MEC-UP cases were identified.

**Results:**

Review of the limited cases suggest many clinicians use positron emission tomography-computed tomography (PET-CT) in addition to panendoscopy and targeted biopsies with consideration for diagnostic tonsillectomy in diagnostic work-up.

**Conclusion:**

Like other salivary gland cancers, primary therapeutic surgical resection is recommended with low threshold for adjuvant radiotherapy to regions at high risk for harbouring the primary malignancy, especially in cases of high-grade histopathology.

## Introduction

Mucoepidermoid carcinoma (MEC) is the most common salivary gland malignancy, characterized by a mixed histological pattern of epidermoid, mucous-producing and intermediate cells.^1^^–^^3^ While often found in the parotid glands, primary tumours can arise in any major or minor salivary glands.^4^^,^^5^ Prognosis in MEC is variable, with studies showing five-year survival rates between 87 and 98 per cent and 22 and 67 per cent in low-grade and high-grade tumours, respectively.^6^^–^^9^

Cancers of unknown primary (CUP) present as metastatic lesions without any known primary site despite investigative work-up.^10^ CUPs represent 3–5 per cent of all malignant epithelial tumours, carry a worse prognosis than their counterparts with known primary and can be aggressive with a poor response to empiric treatment.^11^^–^^14^ They are estimated to comprise around 5 per cent of head and neck cancers, with five-year overall survival rates reported around 30–40 per cent.^15^ Current literature detailing management of head and neck CUP is mostly in the context of squamous cell carcinoma, with little information on the management of MEC-UP available.^16^^,^^17^ Hence, MEC-UP presents a unique clinical challenge, with only a handful of case reports detailing management and outcomes.

To guide clinical decision making in this rare presentation of head and neck cancer, we embarked on the first systematic review of the MEC-UP literature for additional cases to present alongside our two (Appendix 1). This resulted in nine total cases of MEC-UP, the largest number of MEC-UP cases presented in a single article to date.

## Methods

We performed a database search of Medline (Ovid) and Embase from database inception to April 2024 (Appendix 1). This revealed 1560 unique citations that underwent screening and full-text review in duplicate (K.C. and P.S.). We identified five case reports that investigated MEC-UP of the head and neck (Table 1).

**Table 1.** Summary of published cases of MEC-UP in the head and neck

Study	Age and sex	Diagnostic work-up	Site of tumour presentation	Pathology details	Initial staging	Pertinent medical comorbidities	Management	Adjuvant/salvage therapy	Clinical outcome
1. Friedrich *et al*. (2016)^47^	48M	US, MRI, PET-CT, open LN biopsy	Left level II neck mass	Low grade MEC, 2.4cm, 1/3 lymph nodes positive for MEC	TxN1M0	None reported	Ipsilateral MRND	CRT to primary site candidate and neck (details not specified)	No disease recurrence 43-months after primary treatment
2. Prabhu *et al*. (2011)^48^	67M	CT, MRI, FNAB, PE	Right level II neck mass	Low grade MEC, 3.5cm, “extensive” nodal involvement	Not reported	51 PY smoking history	Ipsilateral RND	RT: 60 Gy over six weeks to right neck	Distant metastases within 24 months of primary treatment
3. Ghazali *et al*. (2017)^42^	66M	CT, PET-CT, FNAB, PE	Right level II neck mass	High grade MEC, 3 cm, 51/61 lymph nodes positive for MEC	TxN3M1	Smoking history, history of resected oral tongue CIS	Concurrent CRT (i.e., 70 Gy and weekly cisplatin)	Ipsilateral MRND, contralateral SND	Death 2 months after primary treatment
4. Trosman *et al*. (2014)^49^	43M	CT, PET-CT, open LN biopsy	Right level II neck mass	Intermediate grade MEC, 7cm, multiple lymph nodes with ENE	TxN3M0	None listed	Ipsilateral SND	RT to primary site candidate and right neck (details not specified)	Disease free after 1 year
5. Wani *et al*. (1998)^50^	59F	CT, open LN biopsy, PE	Left level II neck mass	Intermediate grade MEC, 3cm, one positive node	TxN2M0	None listed	Total parotidectomy, ipsilateral MRND	None	Disease free after 1 year
	47M	CT, FNAB, open LN biopsy, PE	Left neck mass	No details reported	TxN3M0	Heavy smoking, alcohol abuse	Primary lesion resection and ipsilateral neck dissection	Brachytherapy (details not specified)	Regional recurrence 9 months after primary treatment
6. Yulian *et al*. (2022)^51^	52M	CT, PET-CT, MRI, US	Right axillary lump 2.5 years post-non MEC-UP in neck	High grade MEC, 3.9 cm, LVI	Not reported	6 PY smoking history	Prior to MEC diagnosis - Primary: Bilateral RND; adjuvant: CRT with 30 fractions and 7 cycles of cisplatin	Axillary dissection	None reported
7. Chalmers *et al*. (2024) (this study)	41F	CT, MRI, PET-CT, FNAB, PE	Left level II neck mass	Low grade MEC, 1.2cm, one positive node excised	TxN1M0	Lyme disease, EBV, HSV2+	Clinical surveillance	None	No documented recurrence 18 months after initial presentation
	65F	CT, MRI, FNAB, PE	Left level II neck mass	Low grade MEC, 3.2cm, 1/19 lymph nodes positive	TxN1M0	Hypothyroidism, hypertension, GERD	Left level I-III and partial IV neck dissection	Did not undergo RT due to clear margins, low grade MEC, no primary site	No documented recurrence 7 months after primary treatment

CRT = chemoradiotherapy; EBV = Epstein–Barr virus; ENE = extranodal extensions; FNAB = fine needle aspiration biopsy; GERD = gastroesophageal reflux disease; HSV = herpes simplex virus; LN = lymph node; LVI = lymphovascular invasion; MEC = mucoepidermoid carcinoma; MEC-UP = mucoepidermoid carcinoma of unknown primary; MRI = magnetic resonance imaging; MRND = modified radical neck dissection; PE = panendoscopy; PET = positron emission tomography; PY = pack-year; RND = radical neck dissection; RT = radiotherapy; SND = selective neck dissection; US = ultrasound.

## Case report

### Case one

A 41-year-old female presented with an asymptomatic left-sided lateral neck mass and an otherwise unremarkable physical exam. She is a never-smoker with a history of Lyme disease, Epstein–Barr virus and herpes simplex virus. Her fine needle aspiration biopsy (FNAB) demonstrated cellular atypia but was negative for carcinoma and so she underwent an excisional biopsy that demonstrated low-grade MEC. She underwent panendoscopy with base of tongue biopsies and ipsilateral diagnostic tonsillectomy that was negative for any malignancy. She was staged as TXN1M0. Her imaging post-excisional biopsy did not demonstrate any additional lesions of concern. Her case was presented at multidisciplinary tumour boards who recommended ongoing oncological surveillance with consideration for neck dissection if any changes were to arise. The patient decided against an ipsilateral neck dissection and instead chose regular surveillance. She has had subsequent ultrasound, MRI and PET-CT that did not demonstrate any pathological features consistent with a primary site. Presently, she has not developed any recurrent disease or lesions consistent with a primary tumour within 18 months of her initial presentation.

### Case two

**Figure 1.**
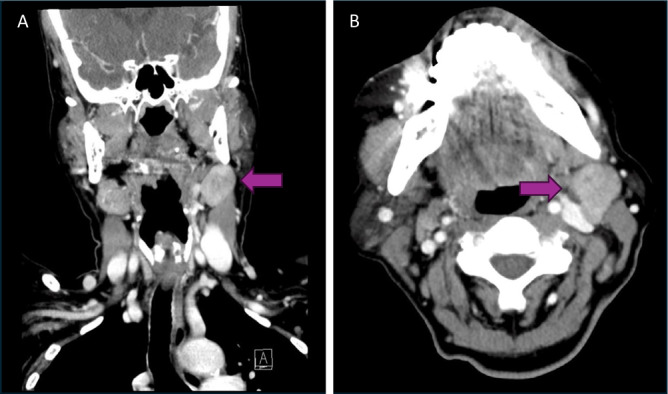
CT neck with contrast in coronal (A) and transverse (B) demonstrating a 2.2 x 2.3 x 3.2 cm heterogeneous mass between left submandibular gland and left SCM.

**Figure 2.**
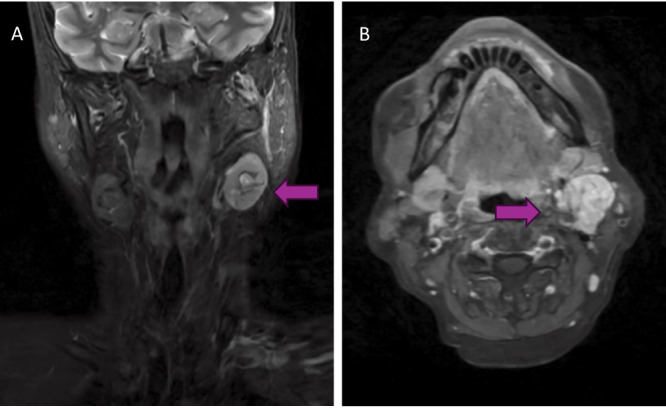
MRI neck with gadolinium contrast in coronal (A) and axial (B) demonstrating the same left sided level IIA neck mass measuring alongside a small mixed solid/cystic lymph node.

A 65-year-old female presented with an asymptomatic left-sided neck mass. Three years prior to presentation, she underwent a FNAB of this neck mass outside of Canada that suggested Warthin’s tumour. She is a never-smoker, and her past medical history included hypothyroidism, hypertension and gastroesophageal reflux disease (GERD). Due to continued growth, the mass was investigated by repeat FNAB, which showed atypical cells. CT neck with contrast showed a heterogeneous high-density mass in the left neck (Figure 1). A subsequent core needle biopsy indicated low-grade MEC, and an MRI neck with gadolinium enhancement showed an intermediate-enhancing lesion in the left upper cervical area and a small mixed solid/cystic lymph node (Figure 2). Panendoscopy revealed no obvious mucosal lesions and left tonsillectomy and direct biopsy of the left, central and right tongue base were negative for malignancy. She was staged as TXN1M0.

Primary management was ipsilateral selective neck dissection of levels I–III with partial level IV and resection of peri-facial nodes and the tail of the parotid gland. Surgical pathology showed 1/19 lymph nodes were positive for MEC alongside negative margins and no extranodal extension. The patient was referred for consideration for adjuvant radiotherapy (RT), but due to the lack of high-risk pathological features, the team instead opted for ongoing surveillance. Presently, she does not have any disease recurrence within 7 months of primary treatment.

## Discussion

To the best of our knowledge, this report is the largest published series of patients with MEC-UP of the head and neck (Table 1). Across these studies, the median age at presentation was 54 years (range: 43–67 years), and most patients were male, presenting with asymptomatic neck masses. We found that 44 per cent of these patients had a history of smoking, but smoking status remains an unclear risk factor in MEC despite being associated with other salivary gland malignancies.^18^^,^^19^ In one case, we found that MEC was diagnosed three years following a FNAB suggestive of Warthin’s tumour. Warthin’s tumour rarely presents outside of the parotid gland, but cases of MEC developing in a Warthin’s tumour have been reported.^20^^–^^22^ There are also described cases of Warthin’s tumour-like MEC, which are MEC tumours that possess cellular features similar to Warthin’s tumour.^23^ These uncommonly described tumours may represent malignant transformation of Warthin’s tumour, or “tumour-to-tumour” metastasis from a distant primary, which is a phenomenon that has been described in other cancers.^23^^,^^24^ Zhang and colleagues suggested that testing for the MAML2 gene via fluorescence in situ hybridization can identify Warthin’s tumour-like MEC and prevent cytological misdiagnosis.^25^ In summary, head and neck MEC-UP often presents as an asymptomatic neck mass wherein initial diagnosis is often guided by FNAB with consideration for core needle and/or open biopsy if FNAB results are unclear.

Further diagnostic work-up of the primary site in MEC-UP should be modelled after head and neck mucosal CUP. We propose that clinicians consider employing imaging modalities such as CT, MRI and fluorodeoxyglucose (FDG)-PET/CT, in addition to panendoscopy and biopsies of any suspicious mucosal sites. In our review, four studies performed an FDG-PET/CT scan to help localize and stage the primary site following initial work-up with CT and MRI. In oncology, FDG-PET/CT scans are recommended to identify and stage CUPs for any anatomic region.^26^^–^^29^ Moreover, FDG-PET/CT scans are better than CT alone in detecting primary sites, nodal deposits and distant metastases in salivary gland cancer.^30^^,^^31^ Hence, we recommend that clinicians consider FDG-PET/CT in cases of suspected MEC-UP when CT and MRI do not identify any sites suspicious for a primary cancer. Diagnostic tonsillectomy is often considered for head and neck mucosal CUP.^32^^,^^33^ Although rare, authors have reported primary MEC affecting minor salivary glands within subsites of the oropharynx, including the palatine tonsils.^34^^–^^37^ Hence, clinicians should consider panendoscopy, targeted biopsies and diagnostic ipsilateral tonsillectomy for MEC-UP of the head and neck, especially if any suspicious areas are noted on diagnostic work-up.

Primary salivary gland malignancies are typically treated via primary surgery addressing the primary site and any nodal metastases with indications for adjuvant treatment reserved for high-risk pathological features.^9^^,^^38^ When accounting for high-risk pathological features, survival in salivary gland MEC is associated with histological grade, with five-year survival ranging from 26 per cent to 95 per cent in high and low histological grade disease, respectively.^39^^,^^40^ In MEC-UP, we found neck dissection to extirpate any gross nodal disease was most often performed as primary treatment.^41^ One study reported primary chemoradiotherapy (CRT) followed by salvage neck dissection for a patient presenting with distant metastases who did succumb to his advanced disease within two months of primary treatment.^42^ The use of adjuvant RT in MEC varies based on pathological features and grade of disease, with one case series of MEC reported an overall rate of RT of 41 per cent, where most low-grade disease was treated with surgery alone and most high-grade disease was treated with surgery and adjuvant RT.^43^ This is supported by another case series, which reported the rate of adjuvant RT in salivary gland MEC as 7 per cent in low-grade disease and 70 per cent in high-grade disease.^44^ Studies suggest improved locoregional control with adjuvant RT in MEC patients with high histological grade, positive surgical margins, peri-neural invasion, and/or in advanced stage disease, but this remains controversial.^9^^,^^38^^,^^43^^,^^45^ Most MEC-UP patients in this series received adjuvant CRT, which was typically indicated due to advanced nodal disease.^42^^,^^46^^–^^50^ While no difference in overall survival has been shown in MEC patients receiving adjuvant RT versus CRT, studies suggest that adjuvant CRT confers a greater locoregional control, especially in those with high-risk pathological features.^9^^,^^40^^,^^51^ In cases of MEC-UP, survival may be worsened due to the lack of treatment to the primary site, so, clinicians should have a low threshold for recommending adjuvant RT to affected nodal sites and sites at risk for harbouring the MEC primary site.^52^

- MEC-UP is a head and neck cancer that is poorly described in the literature
- We detail the largest number of cases and management of MEC-UP through the introduction of two cases and a review of the literature
- Work-up of MEC-UP tends to include PET-CT, panendoscopy, targeted biopsies and consideration for diagnostic tonsillectomy
- Primary management of MEC-UP is complete surgical excision
- There is a lower threshold for consideration of adjuvant RT or CRT than in salivary gland malignancies with known primary
- MEC-UP outcomes may be worse due to incomplete identification and removal of a primary site, if one exists

## Conclusion

Herein, we reported the largest review of MEC-UP of the head and neck, which is a rare but challenging surgical diagnosis. From the cases reported, clinicians perform PET-CT in addition to panendoscopy and targeted biopsies with consideration of diagnostic tonsillectomy. As with other salivary gland cancers, primary surgical resection of gross disease is the most common treatment with low threshold for considering adjuvant RT to regions at risk for harbouring the primary malignancy, especially in cases of high-grade histopathology.

## Supporting information

Chalmers et al. supplementary material Chalmers et al. supplementary material

## Supplementary material

The supplementary material for this article can be found at https://doi.org/10.1017/S0022215124002147.
