# Supplementary material for: Mucoepidermoid carcinoma of unknown primary in the head and neck: a case report and review of the literature
Source: J Laryngol Otol. 2025 Jun;139(6):521–5. doi: 10.1017/S0022215124002147 (PMC12303715; doi:10.1017/S0022215124002147)
Supplement: Chalmers et al. supplementary material [file S0022215124002147sup001.docx]

Appendix A: Databases searches for literature review

Medline Search

Simplified:

| 1. | mucoepidermoid carcinoma.mp. or Carcinoma, Mucoepidermoid/ |
| --- | --- |
| 2. | Neoplasms, Unknown Primary/ |
| 3. | unknown primary cancer.mp. |
| 4. | cancer of unknown primary.mp. |
| 5. | 2 or 3 or 4 |
| 6. | 1 and 5 |

Extensive:

| 1. | Carcinoma, Mucoepidermoid/ |
| --- | --- |
| 2. | Mucoepidermoid Tumor/ |
| 3. | Mucoepidermoid carcinoma.mp. |
| 4. | Mucoepidermoid tumor.mp. |
| 5. | 1 or 2 or 3 or 4 |
| 6. | Neoplasms, Unknown Primary/ |
| 7. | unknown primary.mp. |
| 8. | Unknown primary tumor.mp. |
| 9. | Unknown primary site.mp. |
| 10. | Occult primary cancer.mp. |
| 11. | Occult primary tumor.mp. |
| 12. | Metastatic carcinoma of unknown origin.mp. |
| 13. | Cancer of unknown origin.mp. |
| 14. | Uncertain primary site.mp. |
| 15. | 6 or 7 or 8 or 9 or 10 or 11 or 12 or 13 or 14 |
| 16. | 5 and 15 |
| 17. | (Head and neck cancer).mp. [mp=title, book title, abstract, original title, name of substance word, subject heading word, floating sub-heading word, keyword heading word, organism supplementary concept word, protocol supplementary concept word, rare disease supplementary concept word, unique identifier, synonyms, population supplementary concept word, anatomy supplementary concept word] |
| 18. | (Head and neck neoplasm).mp. [mp=title, book title, abstract, original title, name of substance word, subject heading word, floating sub-heading word, keyword heading word, organism supplementary concept word, protocol supplementary concept word, rare disease supplementary concept word, unique identifier, synonyms, population supplementary concept word, anatomy supplementary concept word] |
| 19. | (Head and neck malignancy).mp. [mp=title, book title, abstract, original title, name of substance word, subject heading word, floating sub-heading word, keyword heading word, organism supplementary concept word, protocol supplementary concept word, rare disease supplementary concept word, unique identifier, synonyms, population supplementary concept word, anatomy supplementary concept word] |
| 20. | (Head and neck metastasis).mp. [mp=title, book title, abstract, original title, name of substance word, subject heading word, floating sub-heading word, keyword heading word, organism supplementary concept word, protocol supplementary concept word, rare disease supplementary concept word, unique identifier, synonyms, population supplementary concept word, anatomy supplementary concept word] |
| 21. | Head metastasis.mp. |
| 22. | Neck metastasis.mp. |
| 23. | (Head and neck tumor).mp. [mp=title, book title, abstract, original title, name of substance word, subject heading word, floating sub-heading word, keyword heading word, organism supplementary concept word, protocol supplementary concept word, rare disease supplementary concept word, unique identifier, synonyms, population supplementary concept word, anatomy supplementary concept word] |
| 24. | "Head and Neck Neoplasms"/ |
| 25. | Salivary Glands/ |
| 26. | Salivary gland.mp. |
| 27. | Salivary Gland Neoplasms/ |
| 28. | Salivary gland neoplasms.mp. |
| 29. | Salivary gland tumor.mp. |
| 30. | Salivary gland cancer.mp. |
| 31. | Parotid Gland/ |
| 32. | Parotid gland.mp. |
| 33. | Submandibular Gland/ |
| 34. | Submandibular gland.mp. |
| 35. | Sublingual Gland/ |
| 36. | Sublingual gland.mp. |
| 37. | Neck cancer.mp. |
| 38. | Neck tumor.mp. |
| 39. | Mouth Neoplasms/ |
| 40. | Mouth neoplasm.mp. |
| 41. | Mouth cancer.mp. [mp=title, book title, abstract, original title, name of substance word, subject heading word, floating sub-heading word, keyword heading word, organism supplementary concept word, protocol supplementary concept word, rare disease supplementary concept word, unique identifier, synonyms, population supplementary concept word, anatomy supplementary concept word] |
| 42. | 17 or 18 or 19 or 20 or 21 or 22 or 23 or 24 or 25 or 26 or 27 or 28 or 29 or 30 or 31 or 32 or 33 or 34 or 35 or 36 or 37 or 38 or 39 or 40 or 41 |
| 43. | 5 or 15 |
| 44. | 42 and 43 |
| 45. | Case Reports/ |
| 46. | case report.mp. |
| 47. | 45 or 46 |
| 48. | 44 and 47 |
| 49. | mucoepidermoid.mp. |
| 50. | 48 and 49 |

Appendix B:

Embase Search

Simplified

| 1. | mucoepidermoid carcinoma.mp. or mucoepidermoid tumor/ |
| --- | --- |
| 2. | mucoepidermoid.mp. |
| 3. | cancer of unknown primary.mp. or "cancer of unknown primary site"/ |
| 4. | occult.mp. or occult cancer/ |
| 5. | 1 or 2 |
| 6. | 3 or 4 |
| 7. | 5 and 6 |

Extensive

| 1. | mucoepidermoid tumor/ |
| --- | --- |
| 2. | Mucoepidermoid tumor.mp. |
| 3. | mucoepidermoid carcinoma.mp. |
| 4. | mucoepidermoid cancer.mp. |
| 5. | "cancer of unknown primary site"/ |
| 6. | Cancer of unknown primary site.mp. |
| 7. | unknown primary neoplasm.mp. |
| 8. | Unknown primary.mp. |
| 9. | unknown primary tumor.mp. |
| 10. | unknown primary site.mp. |
| 11. | unknown primary cancer.mp. |
| 12. | unknown primary malignancy.mp. |
| 13. | unknown primary malignancy origin.mp. |
| 14. | CUP.mp. |
| 15. | occult primary cancer.mp. |
| 16. | occult primary tumor.mp. |
| 17. | metastatic carcinoma of unknown origin.mp. |
| 18. | cancer of unknown origin.mp. |
| 19. | uncertain primary site.mp. |
| 20. | 1 or 2 or 3 or 4 |
| 21. | 5 or 6 or 7 or 8 or 9 or 10 or 11 or 12 or 13 or 14 or 15 or 16 or 17 or 18 or 19 |
| 22. | 20 or 21 |
| 23. | (head and neck cancer).mp. [mp=title, abstract, heading word, drug trade name, original title, device manufacturer, drug manufacturer, device trade name, keyword heading word, floating subheading word, candidate term word] |
| 24. | (head and neck neoplasm).mp. [mp=title, abstract, heading word, drug trade name, original title, device manufacturer, drug manufacturer, device trade name, keyword heading word, floating subheading word, candidate term word] |
| 25. | (head and neck malignancy).mp. [mp=title, abstract, heading word, drug trade name, original title, device manufacturer, drug manufacturer, device trade name, keyword heading word, floating subheading word, candidate term word] |
| 26. | (head and neck metastasis).mp. [mp=title, abstract, heading word, drug trade name, original title, device manufacturer, drug manufacturer, device trade name, keyword heading word, floating subheading word, candidate term word] |
| 27. | (head and neck tumor).mp. [mp=title, abstract, heading word, drug trade name, original title, device manufacturer, drug manufacturer, device trade name, keyword heading word, floating subheading word, candidate term word] |
| 28. | head metastasis.mp. |
| 29. | salivary gland/ |
| 30. | salivary gland tumor/ |
| 31. | salivary gland.mp. |
| 32. | salivary gland neoplasm.mp. |
| 33. | Salivary gland tumor.mp. |
| 34. | salivary gland cancer/ |
| 35. | Salivary gland cancer.mp. |
| 36. | parotid gland/ |
| 37. | parotid gland.mp. |
| 38. | submandibular gland/ |
| 39. | submandibular gland.mp. |
| 40. | sublingual gland/ |
| 41. | sublingual gland.mp. |
| 42. | neck cancer/ |
| 43. | "head and neck cancer"/ |
| 44. | neck cancer.mp. |
| 45. | neck tumor/ |
| 46. | neck tumor.mp. |
| 47. | mouth tumor/ |
| 48. | mouth neoplasm.mp. |
| 49. | 23 or 24 or 25 or 26 or 27 or 28 or 29 or 30 or 31 or 32 or 33 or 34 or 35 or 36 or 37 or 38 or 39 or 40 or 41 or 42 or 43 or 44 or 45 or 46 or 47 or 48 |
| 50. | case report/ |
| 51. | case report.mp. |
| 52. | case study/ |
| 53. | case series.mp. |
| 54. | case study.mp. |
| 55. | otorhinolaryngology/ |
| 56. | otolaryngology.mp. |
| 57. | ENT.mp. |
| 58. | 50 or 51 or 52 or 53 or 54 |
| 59. | 55 or 56 or 57 |
| 60. | 22 and 58 |
| 61. | 59 and 60 |
| 62. | 22 and 49 |
| 63. | 58 and 62 |
| 64. | 59 and 63 |
| 65. | case.mp. |
| 66. | cohort.mp. |
| 67. | case study/ |
| 68. | case study.mp. |
| 69. | case report.mp. |
| 70. | cohort analysis/ |
| 71. | 65 or 66 or 67 or 68 or 69 or 70 |
| 72. | 58 or 71 |
| 73. | 62 and 72 |
| 74. | mucoepidermoid.mp. |
| 75. | 73 and 74 |
